# Toward practical transparent verifiable and long-term reproducible research using Guix

**DOI:** 10.1038/s41597-022-01720-9

**Published:** 2022-10-04

**Authors:** Nicolas Vallet, David Michonneau, Simon Tournier

**Affiliations:** 1 Université de Paris, INSERM U976, F-75010 Paris, France; 2 Hematology Transplantation, Saint Louis hospital, 1 avenue Claude Vellefaux, 75010 Paris, France; 3 Université de Paris, INSERM US53, CNRS UAR 2030, Saint Louis Research Institute, 1 avenue Claude Vellefaux, 75010 Paris, France

**Keywords:** Research management, Computational platforms and environments, Software

## Abstract

Reproducibility crisis urge scientists to promote transparency which allows peers to draw same conclusions after performing identical steps from hypothesis to results. Growing resources are developed to open the access to methods, data and source codes. Still, the computational environment, an interface between data and source code running analyses, is not addressed. Environments are usually described with software and library names associated with version labels or provided as an opaque container image. This is not enough to describe the complexity of the dependencies on which they rely to operate on. We describe this issue and illustrate how open tools like Guix can be used by any scientist to share their environment and allow peers to reproduce it. Some steps of research might not be fully reproducible, but at least, transparency for computation is technically addressable. These tools should be considered by scientists willing to promote transparency and open science.

## Introduction

Transparency is a central concept in science. For instance, validation of experimental data by scientific community relies on the ability to reproduce or replicate^1^ any scientific result by independent teams. Transparency allows peers to draw same conclusions after performing identical steps from the hypothesis to results^1,2^. Knowing that scientists agree on the existence of a significant crisis of reproducibility and replicability^3–5^, there is a need to move toward practices that ensure that every steps are transparent and verifiable.

**Fig. 1:**
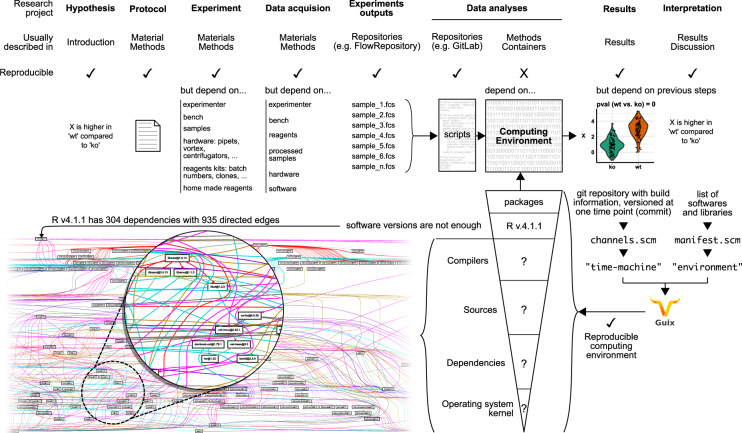
Schematic view of research process and the corresponding methods to ensure reproducibility. Main steps of a research project are currently addressed within the final version of a manuscript. This manuscript can point to several files repositories that host data, scripts or even computing environment in container images. Computing environment is not described enough to ensure reproducibility. Even version numbers are not enough as they cannot describe all dependencies of one software. Likewise, containers reproduce an environment but do not capture all dependencies used for their compilation thus cannot be rebuilt. In this example, a computational environment with R v4.1.1 (without packages) requires 304 binaries all bound by 935 edges. Ideally, the dependency tree should be described to allow reproducibility by any user at any-time. Though this huge list may seem daunting, tools like Guix can rebuilt dependency tree with the list of software used and the path to Git repositories with the all information to build them from scratch. Sharing these two files allow reproduction of computational environment byte-by-byte. For visualization purposes directed acyclic graph represents a cropped view of compiled dependencies of R version 4.1.1. A complete view of R dependency tree is available the GitLab repository with codes to reproduce graphs.

Assuming that the final outcome of a scientific result is summarized in the form of a published article, the publication describes one hypothesis and the related methods to explore such. Then, by reporting a series of results following data analysis, it leads to conclusions about the hypothesis. Firstly, how data are generated is usually detailed in Material and Methods sections: samples preparation, reagents and various used instruments. Secondly, data analysis is commonly also exposed through Material and Methods section and explained by Results section (Fig. 1).

Open research^6^ and FAIR principles^7^ promote, among many other practices, transparency for all the stages. Having full access to critical stages allows to scrutinize the final outcome, nowadays or in some future. It comes in the form of accessible pre-print or open journals or repositories where all material and methods, archived data and source codes of analyses are disclosed. For example, the content is now available through several public archives: pre-print servers, public source code and data repositories, software hosting platforms. Hence, using this framework, one key information is still missing: the interface between generated data, analyses and results. This interface may be summarized as the computational environment: all the software and libraries used to complete the analysis. This computational environment allows to interact and transform the generated data to readable results. However, the computational environment exploited to run the whole chain of analyses is often skipped or incompletely described in scientific reports. Therefore, the result fails to some transparency, verification and reproducibility.

Computational environment employed to explore initial scientific hypothesis may be described with software or library names and their corresponding version. While names and version labels are a step toward transparency, they are insufficient to allow any future user to rebuild the computational environment on which these software are running. This is illustrated, for instance, by reported failures to reproduce analyses^5^ (Fig. 1).

The aim of this paper is twofold: to describe the complexity of the issue to tackle, then to introduce tools that may help to address such issue. We next apply GNU Guix to provide a practical example in the context of  cytometry analyses.

## Results

### The problem: software version is not enough

A software running on any computer is the result of one source code transformed into binary by another software (e.g., compiler). The compiler, also binary, is thus obtained from source by other software, so called built-time dependencies. Hence, running a single software implies a recursive stack of binaries. This is a well-known chicken or the egg problem named bootstrapping. Even after this compilation step, the resulting executable file may also depend on other binaries such as dynamical libraries, so called run-time dependencies. For example, let’s consider the broadly used R language^8^. Once downloaded and installed, regular user just runs the command R. Behind the scene, this simple command involves a large stack. At first, the command is tangled to a shell script which calls the R interpreter. This R interpreter is a program mainly written in the language C, thus it needs to be transformed by one compiler into one executable binary. Hence, although two computers run the exact same version of the source code of the R interpreter, there is no guarantee that the stack of binaries is identical on both computers, consequently, the produced executable binary may not be the same. Then, this executable R interpreter depends on libraries at run-time. Again, there is no guarantee that these libraries are the same on both computers. On the top of that, the shell script requires a binary shell interpreter, and then, all the explanations drawn above also apply.

Therefore, describing the version of source code is already better than nothing, but it does not provide all the useful information for redeploying the computational environment, later or elsewhere. Back to the example, reporting that one analysis was done using both R at version 4.1.1 and the list of R libraries is not a guarantee that from the source code of R at version 4.1.1 and these R libraries, one might be able to complete this very same analysis again. Instead, to be fully transparent and reproducible, the description of the computational environment should not be limited to version labels but should capture the whole computational stack (compilers, built-time and run-time dependencies) (Fig. 1).

Failure to describe the whole computational environment leads to two issues. One is about the level of transparency. If a user is running the same analysis on another computer using another computational stack but using same source code versions and does not get the same results, then is it because of a flaw of the analysis or is it because of a variation of the computational stack? Similarly, how could one inspect for improving tools when the whole computational stack is not described? Then, second is about practical purpose. It seems impossible to manually describe the whole stack by providing only version labels since the number of dependencies required to just run the R interpreter is already huge: 304 compiled dependencies or 793 dependencies from the full origin source codes (Fig. 1).

### Solutions: build computational environments

In this section, we highlight the existing solutions and how they address the problem. Most of them allow to build and run a computational environment, but they lack a feature for being able to re-build later the exact same computational environment.

#### Package managers: keep under control the dependencies

**Fig. 2:**
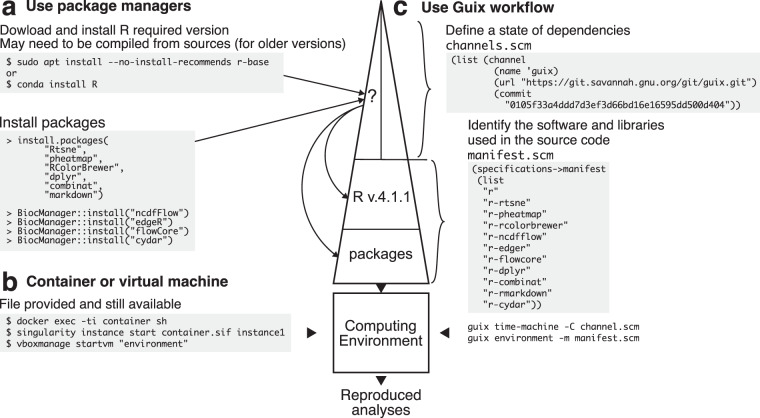
Methods to build a computational environment. (**a**) Package managers handle binary production and installation from a standardized recipe specific to one software. With several command lines users are able to install the version of software and packages required. To build the binary from source, local system dependencies will be used. Here it is near impossible to describe the whole stack of dependencies used by one system to build the software and packages. (**b**) Container or virtual machine capture the whole computational environment independently from host computer system. Here users can run the analyses in an already compiled environment. The dependencies required to build the container are not described within the container. (**c**) Guix workflow uses the list of software and packages combined with a fixed state of dependencies defined in a channel. With these two inputs, Guix build the computation environment. Here, all steps of computing environment compilation are transparent and reproducible.

The aim of the package managers is to handle the binary production and its installation. From a standardized recipe usually specific to one software, the package managers deal with both build-time and run-time dependencies, often satisfying constraints on version labels, and thus ease the distribution of software. Package managers may be for general purpose as operating system (OS)-provided ones or may be oriented as programming language-provided ones (e.g., install.packages from the R language ecosystem) or may be mixed as Conda or BioConda^9^. When reproducing a computational environment using one package manager, the core question is the influence of the host computer system^9^. Assuming the analysis was run on one specific host computer system, then the ability to re-run later on another host is strongly affected by the variation on the two different hosts. Most package managers defeat this criterion (Fig. 2a). Thus, they fail to provide reproducible computational environment.

#### Containers: keep under control the impact of the host

The aim of containers (e.g., Docker^10^, Singularity^11^, or “virtual machines”) is to capture the computational environment independently from the host computer system. They rely on stable low-level, light-weighted interactions with the host. Therefore, the analysis may be reproduced later on another computer system (Fig. 2b). Manual description of all the dependencies is impossible from a practical viewpoint. Instead, the binary container is gathered from binaries usually produced by some package managers. This approach is widely deployed and, at some extent, has successfully been used for reproducing analysis^10,12^.

However, when reproducing an analysis using a container, two core questions need to be addressed: (i) how to inspect the binary container for validating the results^13^, and (ii) how to reproduce later this container. Containers defeat the criterion of long term because it is not practically affordable to retain them at scale. Considering the storage capacity they require, it is not sustainable to attach an analysis to a container, one-to-one. Therefore, long term sustainability implies the ability to reproduce the computational environment, which may, but not only, be distributed using a binary container pack format.

Efforts are deployed toward providing reproducible build of containers. For instance, by using a linter, a software that check programming coding style requirements^14^, to Dockerfiles, as the tool Hadolint^10^. But, because they are fed using a package manager, they often hit the lack of the feature from the very package manager to time travel. Assuming the container is built today, then an attempt to re-build it later requires at least, (i) that the infrastructure behind the package manager is still available, and more importantly, (ii) that the package manager can exploit the infrastructure as it is today but later. Some popular Linux distribution provides snapshots of their state at specific moments, but, to our experience, their use is complex^15^.

#### Online infrastructures: delegate the issue

On a side note, effort is currently being developed to implement interactive scripts within articles^16^ or interactive web-based interface to analyze data (e.g. Cytobank^17^, Omiq, Metaboanalyst^18^, Galaxy^19^). These tools are useful to ease interaction with complex data and to provide a simplified workflow of analyses. However, these do not capture the whole computational environment required to run analyses from the source code. Moreover, it raises the question about the long-term support for the computational infrastructure running these scripts, assuming such infrastructure are fully transparent and verifiable. To our knowledge, these online infrastructures hit the same core question as the ones for package managers and containers.

### Solution: share reproducible computational environment

For being truly long-term reproducible, the solution must capture the whole computational environment, control the complete recursive stack and be able to redeploy anytime. Guix (https://guix.gnu.org) attempts to apply principles from functional programming to package management, work pioneered by Nix (https://nixos.org). The installable binary package is modelled by “pure functions”: the installable binary package corresponds to a process where its output only depends on the inputs (build-time and run-time dependencies)^20^. Therefore, Guix provides a deployment system that ensures reproducibility by design.

The Guix system is implemented using the Scheme language^21^, from the core to the high-level domain-specific language (DSL) for specifying and declaring the configuration. To our opinion, the main difference between the Guix and Nix systems is, for the former a continuity from the high-level declarative configuration to the core which allows to adapt or extend the Guix system, and for the latter, on one hand “Nix expressions” written in a DSL specifically designed to define these “pure functions” (derivation), and on the other hand another language for the core. This difference is slight in practice but emphasized by the data processing workflow engines Guix Workflow Language (GWL) based on Guix and BioNix^22^ based on Nix (https://youtu.be/pwYhPqaUiGg, https://youtu.be/tpLcwfRXL28). Last, to our knowledge, the Nix system does not provide yet a mechanism easing the time travel.

### Reproducible computational environment using Guix

Here, we describe how computational part of the reproducibility cycle is addressed when using Guix. As exposed previously, two components have to be provided: the list of software effectively used and an identifier committing the complete stack.

First, the manifest listing the top-level software or library names effectively used by the analysis is provided in a text file, e.g., named manifest.scm. For instance, this manifest file contains the software or library names which are often already described by some papers. Software are packaged in Guix using a recipe, and this recipe notably points to the location of the upstream sources, provides the version label and lists the dependencies for building and running. If the required tool is not indexed yet by Guix, the missing package may be then locally defined by providing an extra file which describes the recipe to build such tool. Moreover, Guix also provides custom channels feature to ease the exchange of such recipes contributed by the community or dedicated to a particular field. These channels are imperatively versioned and their history is tracked by the Git version control system^23^. Moreover, the recipes provided by default are part of Guix itself and all is versioned using also a Git repository (Fig. 2c).

Second, using the list of dependencies from the recipe, Guix determines a graph modelling the complete stack. Each node represents one single tool and the edges identify the dependency relationship. To fully describe this graph, the user provides a text file, e.g., named channels.scm, recording the web address of the channels, Guix itself and the custom channels if any, and their corresponding revision identifier. The revision identifiers (Git commit hash) for each channel allow to determine without any ambiguity the graph and its exact configuration, and thus they allow to build the computational environment regardless of the state of the underlying host system (Fig. 2c).

**Fig. 3:**
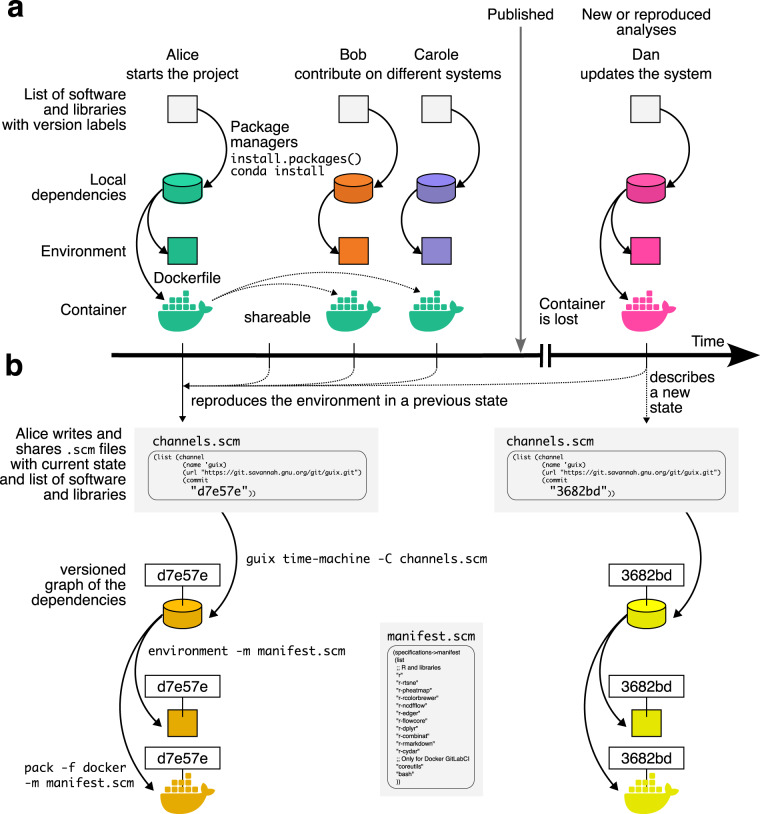
Comparison of workflows in building and sharing environment. (**a**) Multiple contributors may participate in one project. Even with the same list of software and libraries, local dependencies are required to build the environment. Thus, each contributor will use a unique environment that differs from the other ones. To circumvent this issue, one user can build a container and share it. But, the container may be lost in the future. Since systems and dependencies are evolving fast, there is no guarantee that future scientist will be able to build the initial container again. (**b**) Using Guix, the version of the dependencies graph (channel.scm) is described with the list of software and packages (manifest.scm). Any contributor can build the environment or even pack a container at any time. One user can thus travel through time of systems dependencies graph just by changing the channel.scm file and reproduce a lost container or an old environment on which previous analyses were performed. This ensure that reproducibility issues do not come from changings in computational environment.

Based on these two files manifest.scm and channels.scm, an independent user is able, using the subcommands “environment” (or newly renamed “shell”) and “time-machine”, to deploy the exact same computational environment. If the infrastructure requires a container image, Guix has the capacity to directly pack Docker or Singularity images (Fig. 3).

For transparency, it is an encouraged practice^7^ to version these additional manifest.scm and channels.scm files along with the other source files required by the analysis.

Last, when upstream sources are no longer available on their initial web address (URL), Guix provides a bridge to the Software Heritage (SWH) initiative. The mission of SWH is to collect, archive and preserve scientific knowledge residing in source code^24^. Therefore, the computational environment managed by Guix can be deployed regardless of the availability of all initial sources, if these sources have been archived by SWH and upstream is missing, then Guix automatically uses the SWH archive as fallback.

### Case study: cytometry analyses on an environment reproduced by Guix

The Guix workflow is straightforward. It requires two components, the list of required software and packages and the graph modeling the complete stack of dependencies. To illustrate how Guix may be used for reproducible research, we consider a fully open access, open data, open source research article^25^. This example allows us to point out two levels of details: first we present how to implement the Guix workflow at the time of publication; and second, we describe how Guix concretely helps in the reproduction once the analysis published. The details of the specific command lines and the content of each file is reported in the corresponding Gitlab repository.

The paper had been published on May, 2020. Although Ma *et al*. work can be considered as high quality in standards of transparency and open science, several information about their computational environment is lacking. For instance, we could deduce from the reported versions of ncdfFlow (v2.30.1) and flowCore (v.1.50.0) that they have used Bioconductor v3.9 released on the May 3, 2019. However, nothing is said about the version of R itself nor about the version of one R dependency e.g. the linear algebra library OpenBLAS. From our point of view, this illustrates the net about many failures when reproducing: version labels are not enough for capturing the computational environment.

**Fig. 4:**
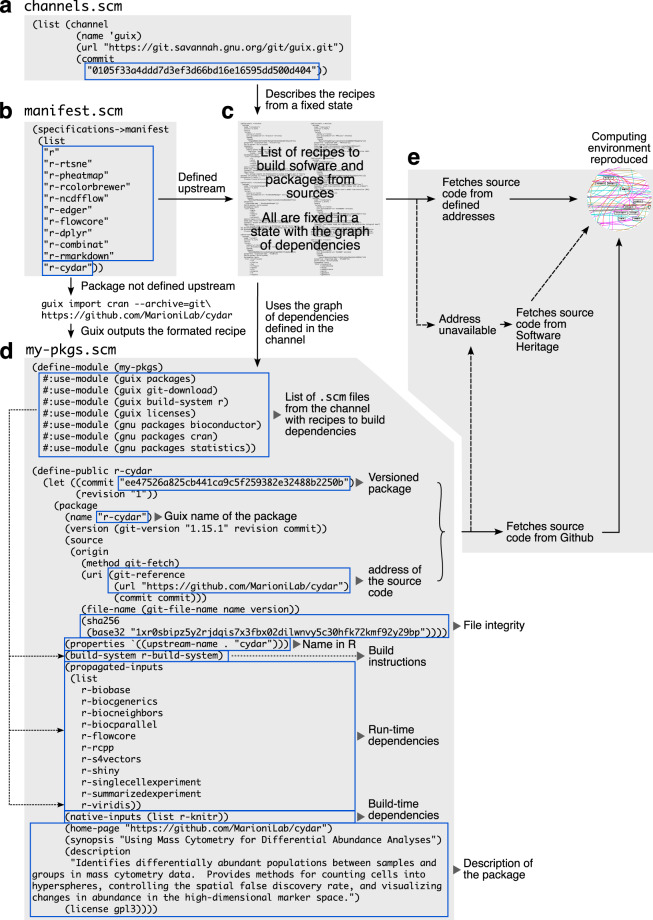
Anatomy of Guix workflow used in the case-study paragraph. (**a**) The channels.scm file defines the channel that will be used to build the graph of dependencies from a list of recipes. This list is fixed at one time in a Git repository. This time-point is accessed with the commit number written in the channel definition. (**b**) The manifest.scm file defines the list of software and packages used in the computational environment. The “r-cydar” package is not defined in the channel thus we have to write the recipe to build it. The command line “guix import” is used to access the recipe from Guix. (**c**) Guix uses the recipes defined in the channel to build the environment, from compilers to the list of software and libraires defined in the manifest.scm. (**d**) All software and packages are defined with the same arguments that are described on the figure. Briefly, a list of dependencies (modules) is defined at the start of the recipe, then the package definition starts with a commit number to version the package. Because the package comes from Git, a git-fetch method is used to gather the source code which is checked for integrity with a hash number (sha256). Next, the method to build the binary is defined with the run-time and build-time dependencies that will be required to run the package. (**e**) The previous steps rely on the hypothesis that URL addresses of the source codes and channels are available to build the environment. In case these addresses are lost, Guix provides a bridge to Software Heritage source code archive to ensure long-term support.

Instead, for reproducing, we need to save the revision of the complete graph modeling all the required dependencies. It is given by the command line “guix describe” which returns the current state of the graph and thus can be saved into the file channels.scm. Namely, this file describes a Git repository containing the recipes to build the required packages and pointing to one specific commit hash (state) (Fig. 4a). For one specific state, Guix is able to populate a computational environment with the exact same binaries.

Back at the time of publication, to our knowledge, the Guix project had not yet packaged the R library ncdfFlow (v2.30.1) but instead the earlier version provided by Guix is v2.32.0. With no loss of generality, and to avoid irrelevant details, we consider Bioconductor v3.10 released on October, 2019. We assume that, at the time of the publication, the authors provided as Materials and Methods the file channels.scm fixing the Guix state at that time.

Next, we describe the software (R libraries) used for the analyses in the file manifest.scm (Fig. 4b,c). Note that the package “r-cydar” is not part of the package collection that Guix provides by default. We thus need to extend the package collection and describe by ourselves the recipe to build this package from its sources hosted on Github. Guix provides facilities to obtain such recipe (https://guix.gnu.org/manual/devel/en/guix.html#Invoking-guix-import) (Fig. 4d). The recipe we used can be found within the file “my-pkgs.scm” in the directory “my-pkgs”.

From these two files manifest.scm and channels.scm, any independent observer can now reproduce the computational environment of interest at any time. Using the command line “guix time-machine -C channels.scm”, Guix instantiates the graph of the dependencies at the state specified by the file “channels.scm” and thus it builds the exact same computational environment specified by the file “manifest.scm”.

Last, a container is sometimes required or the computations need to run in a foreign infrastructure where Guix is not running. Guix is able to pack the binaries and return a container (Docker or Singularity). Herein, the container is transparent and reproducible since it is built using the two files described above. Moreover, the binary container may also be optionally self-contained and hold an internal reference to Guix: when extracted restore the content of the files “channels.scm” and “manifest.scm”. As a proof of concept for inspection, we partially run the analysis using Gitlab.com runners as foreign infrastructure, partially because we applied a downsamplig to reduce the size of the data in order to satisfy the resource offered on this public Gitlab instance.

Using this framework, the environment as it was used at time of publication is saved and can be redeployed in other systems or in the future. If the addresses of source code are unavailable, Guix automatically will fetch source code from SWH (Fig. 4e). This last feature allows long-term reproducibility of computational environment.

The main challenges for reproducing the output from the markdown file were not related to software but to data access. Data had to be fetched manually because of the lack of public application programming interface (API) on Flowrepository. In addition, Flowrepository does not provide file integrity check so there is no guarantee that the repository remained unchanged after the publication.

## Discussion

Computational reproducibility is not only a matter of transparency but also a matter of backward compatibility. Hardware and software are evolving fast. Old digital supports are no more compatible with current technologies. Likewise, source codes of analyses from 10 years ago are difficult to reproduce nowadays^26^.

Here we described how Guix, a package manager, may be used in daily practice to perform analyses on transparent and reproducible computational environment. One of the main advantages of Guix is its backward compatibility. Unexpectedly, Guix development provided a practical example of such software evolution and how potential incompatibilities may be tackled. While we wrote this manuscript, a new subcommand, “shell”, that aims at replacing the “environment” one was introduced (https://guix.gnu.org/en/blog/2021/from-guix-environment-to-guix-shell). Reproducibility of computation from the source code written for this manuscript is guaranteed by the subcommand “time-machine”. Although the subcommand “environment” is disappearing, by pointing to the older revision in which this subcommand is available, Guix is able to build an old version of itself. Then, because this old version of Guix capture the graph of dependencies, it becomes simple to instantiate again the exact same computational environment as we demonstrated when reproducing one paper from two years ago.

Guix implementation is straightforward for users used to command lines interactions. Guix already support more than 20,000 packages and more are regularly added. With only two text files, users can define their computational environment. For most users, the state of all the dependencies are usually specified at the beginning of a project and consists on describing the revision of Guix (commit number). This can be achieved by running one command line that do not require knowledge on Guix Git repository. Readers can access the “R_workflow” directory of our Git repository to start using Guix to build a simple R environment and create their first “channels.scm” and “manifest.scm” files. Moreover, the required tools are just listed in a file and the missing one, if any, can be locally added. Compared to containers, the Guix approach is more flexible and transparent. The environment is not fixed and can evolve alongside with the project without requiring to build new container when new tools must be implemented.

There are several limitations to Guix. The first one is that Guix operates only the Linux kernel. To our knowledge, the full control of the build-time environment is the key to success in reproducible research and such fined-grained control is only available using low-level Linux kernel features requiring special privileges. This limitation about running on the Linux operating system is rooted in solving the chicken or the egg problem of bootstrap (the deep roots of the graph). This limitation is counterbalanced by the fact that Guix requires root access only in the installation process and then any user is able to run Guix commands without special privileges. Furthermore, users without root access or who are not using Linux, can still run the transparent and reproducible container images generated with Guix. Another limitation is that proprietary software are poorly not supported by Guix and pipelines requiring their use cannot be reproduced in that way. This limitation is strongly moderated by the fact that opaque proprietary software is not transparent and thus does not support open research.

The availability of data, source code, scripts, paper, material and methods are necessary requirements for open research but not a sufficient condition for inspecting such research. For instance, their availability tends to decrease after being reported in an article^27,28^. Open access implicitly implies the resilient lookup. It asks, for one about robust identifier and integrity verification, and for two about distributed workload. This latter is out of our scope and it refers to addressing the link rot phenomenon by designing other networks, such as IPFS (https://ipfs.io), GNUnet (https://www.gnunet.org/en/index.html) or RINA (http://csr.bu.edu/rina/index.html). For the former, intrinsic identifiers, which only depend the content itself, opposed to extrinsic identifier attributed by an external authority, aims at providing persistent, unforgettable and verifiable systems. For example, a version of source code referred by a label name is extrinsic and the identification is not always straightforward because this label is not necessarily unique or immutable. On the other hand, Git commit hash summarizes the content itself and thus identifies the source code without any ambiguity (modulo hash function collision). Other examples of intrinsic identifier for citing software are used by the Software Heritage source code archive and discussed in detail by Alliez *et al*.^29^. Long-term open research requires resilient open access based on such intrinsic identifiers.

Reproducibility of the computational environment is driven by transparency. To be fully reproducible one computational environment must be defined by (i) the source code of the software and libraries used (ii) the various options or configuration are required at build-time or run-time and (iii) the complete graph that fixes all the dependencies. Package manager like Nix^22^ and Guix are designed around these three requirements. By using Nix or Guix, scientists are now able to easily share their computational environment. This will allow contemporary and future peers to use the same pipeline of analyses in the exact same computational conditions. The issue of dependencies is not addressed by package managers that will use the locally installed dependencies to build the environment. Containers or virtual machines addresses this issue by providing an already packed environment but they do not provide any information on how the image was build. Thus, the image is not reproducible because of lack of transparency inherent to container compilation.

To conclude, all the steps of the research may not be exactly reproducible. Notably, generating data from experiment may vary for many reasons: sample availability, reagent batches, missing essential piece of information on protocol or simply for financial reasons. At least, the transparent computational part of the reproducible cycle is technically addressable. Guix provides features that allow scientists to share their computational environment, so that, coupled to open data, every computational analysis may be checked and anyone has the capacity to fully inspect them at any time. Guix paves the way and should be considered by scientists willing to promote transparency and open research.

## Methods

Methods and step-by-step procedures to reproduce environments are detailed in out Gitlab repository: https://gitlab.com/nivall/guixreprodsci (Tag: v1.0-pre2) and the version at submission time is archived on Software Heritage at https://archive.softwareheritage.org/swh:1:snp:eb790762a716cb8541a96636ed08659955dc2b15.

## Data Availability

Data used to reproduce cytometry analyses were obtained from FlowRepository https://flowrepository.org/id/FR-FCM-Z2CP.

## Code availability

All source codes are available on our Gitlab repository: https://gitlab.com/nivall/guixreprodsci (Tag: v1.0-pre2) and the version at submission time is archived on Software Heritage at https://archive.softwareheritage.org/swh:1:rev:707f00afef8f6ef1f29a7a4c961dd714f82833f5.
